# Open transcatheter double valve-in-valve replacement for degenerated bioprostheses on the arrested heart

**DOI:** 10.1093/ehjcr/ytad617

**Published:** 2023-12-18

**Authors:** Can Gollmann-Tepeköylü, Johannes Holfeld, Felix Naegele, Michael Grimm, Nikolaos Bonaros

**Affiliations:** Department of Cardiac Surgery, Medical University of Innsbruck, Anichstrasse 35, A-6020 Innsbruck, Austria; Department of Cardiac Surgery, Medical University of Innsbruck, Anichstrasse 35, A-6020 Innsbruck, Austria; Department of Cardiac Surgery, Medical University of Innsbruck, Anichstrasse 35, A-6020 Innsbruck, Austria; Department of Cardiac Surgery, Medical University of Innsbruck, Anichstrasse 35, A-6020 Innsbruck, Austria; Department of Cardiac Surgery, Medical University of Innsbruck, Anichstrasse 35, A-6020 Innsbruck, Austria

**Keywords:** Valve-in-valve, Transcatheter double valve replacement, Bioprosthesis degeneration

## Abstract

**Background:**

Failing bioprosthesis is an emerging issue due to (i) a shift towards liberal bioprosthesis implantation instead of mechanical prosthesis and (ii) an ageing population. Management of high-risk patients with bioprosthesis degeneration remains challenging.

**Case summary:**

An 82-year-old patient with history of aortic and mitral valve replacement six years before presents with severe dyspnoea. Echocardiograpic assessment reveals (i) structural valve degeneration of the mitral prosthesis (severe stenosis and regurgitation) with concomitant major annular calcifications and (ii) structural valve degeneration of the aortic prosthesis with low-flow, low-gradient restenosis. Due to mitral annular calcifications, the risk of double valve re-replacement and the age of the patient conventional reoperation was deemed very high. The patient is evaluated for transapical double valve implantation. This option is rejected due to the high risk of left ventricular outflow obstruction. The patient is treated with an open transcatheter double valve-in-valve procedure at the following sequence: leaflet resection of the mitral bioprosthesis, mitral valve implantation and fixation under direct view, leaflet resection of the aortic bioprosthesis, and valve frame cracking and aortic valve implantation under direct view. Post-bypass echocardiography shows neither left ventricular outflow tract obstruction nor paravalvular leak or prosthesis dysfunction. The patient is extubated on the first post-operative day and transferred to normal care unit.

**Discussion:**

Open transcatheter double valve-in-valve replacement for degenerated bioprostheses on the arrested heart might be a valuable alternative to treat selected high-risk patients with bioprosthetic valve degeneration.

**Learning points**

- Evaluation for double valve reoperation
- Open transcatheter valve implantation
- Techniques to avoid left ventricular outflow tract obstruction in mitral valve-in-valve implantation

## Introduction

Over 200 000 heart valves are implanted annually worldwide. Improved durability and the evolution of transcatheter techniques have caused a shift towards a more liberal use of bioprosthetic heart valves (BHV) instead of mechanical valves.^1^ Despite excellent haemodynamic performance and no need for oral anticoagulation (OAC), BHV are prone to structural valve deterioration (SVD). The glutaraldehyde-fixed xenografts evoke a host immune response resulting in progressive valve destruction with pannus growth, leaflet fibrosis, and micro-calcifications causing haemodynamic deterioration and prosthesis failure.^2^ Predictors for SVD include the size of the used valve prosthesis, patient-prosthesis mismatch (PPM), diabetes, smoking, dyslipidaemia, renal function, the oral anticoagulation regime, and the patient’s age at implantation.^3^ Structural valve deterioration is defined according to the European Association of Cardiovascular Imaging Guidelines as (i) an increase in mean gradient ≥ 5 mmHg for mitral prosthetic valves and >20 mmHg for aortic prosthetic valves (possible SVD) or ≥10 mmHg for mitral prosthetic valves and ≥35 mmHg for aortic prosthetic valves (significant SVD) during follow-up, with a concomitant decrease in valve orifice area and abnormal morphology of the prosthetic leaflets and (ii) presence of new transprosthetic regurgitation.^4,5^

Management of patients with SVD remains challenging, as perioperative mortality after reoperation is significantly increased, especially in elder multimorbid patients, and is as high as 13.8% hospital mortality.^6^ Although there are only a few case reports, transcatheter valve-in-valve replacement might be a feasible option for selected high-risk patients.^7^

## Summary figure

**Figure:**
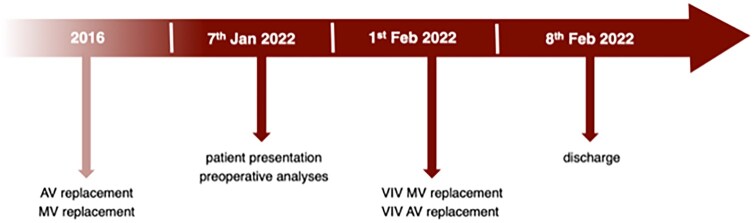


## Case presentation

**Figure 1:**
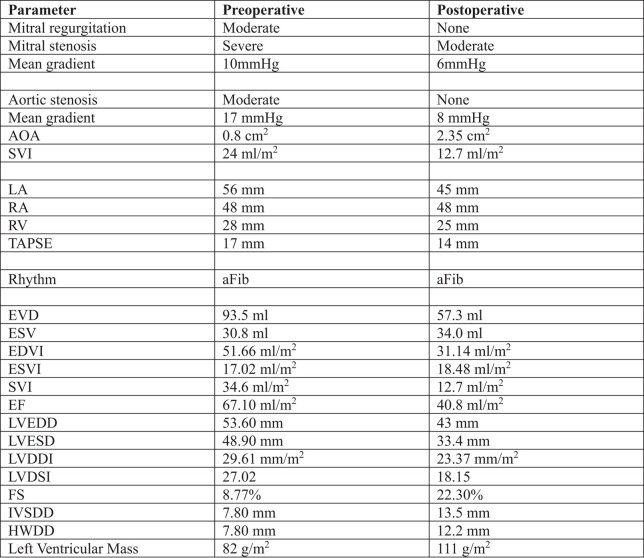
Pre- and post-operative transthoracic echocardiography findings. aFib, atrial fibrillation; AOA, aortic valve area; EDVI, end-diastolic volume index; EF, ejection fraction; ESV, end-systolic volume; ESVI, end-systolic volume index; EVD, end-diastolic volume; FS, fractional shortening; HWDD, posterior wall end-diastolic diameter; IVSDD, interventricular septal end-diastolic diameter; LA, left atrium; LVEDD, left ventricular end-diastolic diameter; LVDDI, left ventricular diastolic diameter index; LVDSI, left ventricular systolic diameter index; LVESD, left ventricular end-systolic diameter; RA, right atrium; RV, right ventricle; SVI, stroke volume index; TAPSE, tricuspid annular plane systolic excursion.

An 82-year-old patient presents with shortness of breath and weakness during exertion and rest. His symptoms have been progressive over the last six months. He was previously active, going for regular walks and hikes but is now home bound, significantly affecting his wellbeing. Physical examination reveals a patient with reduced general condition, i.e. NYHA III–IV. There are no signs of congestion, with normal respiratory sounds. A hemi-sternotomy scar is visible on the patient’s chest. Cardiac auscultation reveals a systolic aortic murmur. The patient has a history of aortic valve (AV) replacement with a 21 mm Carpentier Edwards Magna Ease bioprosthesis and mitral valve (MV) replacement using a 27 mm St. Jude Medical bioprosthesis six years before. By then, he had severe aortic stenosis with concomitant mitral stenosis and massive mitral annular calcifications (*Figure 1*). Transthoracic echocardiographic assessment reveals an excentric severe transvalvular mitral regurgitation with a vena contracta of 6 mm and concomitant severe stenosis (diastolic mean pressure gradient 10 mmHg). The aortic valve shows obvious signs of degeneration (calcifications) and a severe low-flow/low-gradient aortic stenosis with a mean pressure gradient of 17 mmHg, an aortic valve orifice area of 0.8 cm^2^, and an indexed stroke volume of 24 mL/m^2^. The left atrium (LA) is dilated (56 mm), while other cardiac dimensions and ejection fraction (EF) are normal. The patient has signs of right ventricular strain with mild pulmonary hypertension (sPAP 45 mmHg). Additional transoesophageal echocardiography is performed to dissect the underlying valve pathologies. It reveals a moderately degenerated AV prosthesis with a non-mobile non-coronary cusp due to calcifications. After a planimetrical assessment, AV stenosis is confirmed with an orifice area of 0.8 cm^2^. Assessment of the MV confirms a distinct, excentric transvalvular mitral regurgitation (EROA 0.54 cm^2^) with concomitant stenosis. For further patient assessment, right- and left-heart catheterizations are performed.

Mean pulmonary capillary wedge pressure is 9 mmHg, mean right atrial pressure is 2 mmHg, mean right ventricular pressure is 2 mmHg, and mean pulmonary artery pressure of 20 mmHg, respectively. The invasive stroke volume index is 21.77 mL/m^2^, and the invasive cardiac output is 3.39 L/min. There are no signs of coronary artery disease. Carotid ultrasound reveals intima-media thickening on both sides with plaques but no haemodynamically relevant stenosis. Spirometry shows regular pulmonary function. The patient is evaluated for a possible double-valve transcatheter procedure. Cardiac CT reveals no kinking, aneurysms, or relevant stenosis in the femoral and iliac arteries. The patient has increased LDH (388 U/L), hs-TropT (18.6 ng/L), NT-proBNP (835 ng/L), and kidney function is normal (glomerular filtration rate > 60 mL/min/1.73 m^2^). Moreover, the patient has permanent atrial fibrillation (CHADS2VASC-Score 5, OAC: rivaroxaban) and a stroke history after conversion. Current ECG confirms atrial fibrillation (109 b.p.m.), with no abnormalities in QRS or QTc.

**Figure 2:**
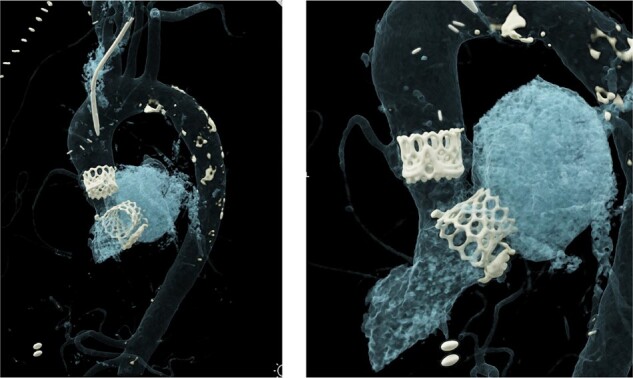
Pre-operative CT evaluation reveals high risk for LVOT obstruction. Cardiac CT shows an angle 112° between LV inflow and outflow. Septal thickness is 7 mm (basal) and 14 mm (midventricular). LVOT diameter is 20 × 27 mm.

The patient is discussed intensively with the heart team. Due to mitral annular calcifications, the risk of double valve re-replacement, and the patient’s age, conventional reoperation risk is deemed very high. This is also reflected by Society of Thoracic Surgeons and EuroScoreII risk assessments, with 6.9% and 6.3%, respectively. Therefore, a transapical double valve implantation is taken into account. This option is rejected due to the high left ventricular outflow obstruction risk. Cardiac CT shows an angle of 112° between LV inflow and outflow. Septal thickness is 7 mm (basal) and 14 mm (midventricular). Left ventricular outflow tract (LVOT) diameter is 20 mm by 27 mm (*Figure 2*).

Moreover, the possibility of a PPM further complicates the decision. Finally, the decision is made to treat the patient with an open transcatheter double valve-in-valve procedure.

**Figure 3:**
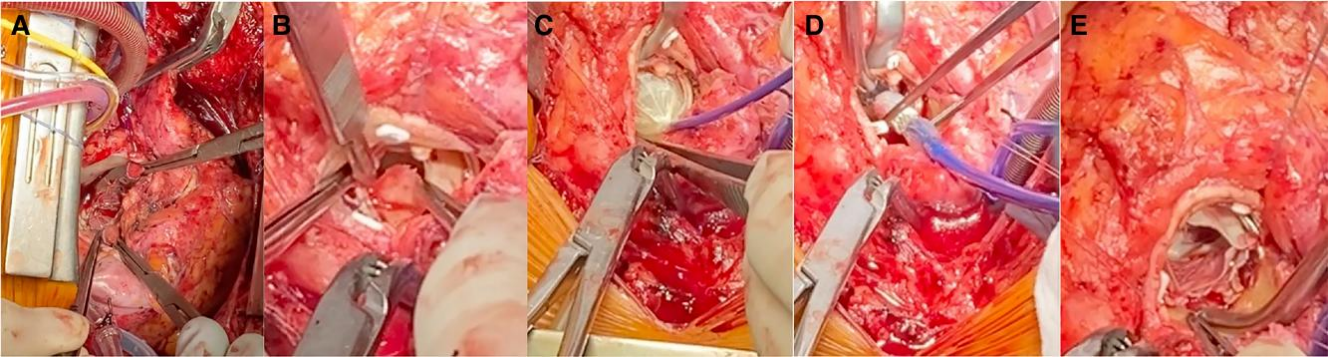
Intraoperative imaging. (*A*) Exposure of the MV prosthesis, (*B*) excision of AV prosthesis leaflets, (*C*) cracking of the prosthesis frame, (*D*) open transcatheter aortic valve implantation (TAVI) implantation in AV position, (*E*) deployed transcatheter valve in AV position.

After median re-sternotomy and installation of cardiopulmonary bypass, the MV is exposed via a left atrial roof incision, and the superior vena cava is disconnected from the heart (*Figure 3A*). The high risk of conventional re-replacement of the mitral valve prosthesis due to the massive mitral annular calcification (MAC) can be confirmed after situs inspection. To avoid obstruction of the left ventricular outflow, the leaflets of the mitral bioprosthesis are resected. Subsequently, three sutures are placed at the ring of the degenerated mitral bioprosthesis corresponding to the three commissures. Finally, a 26 mm Edwards Sapien 3 valve is implanted under controlled expansion and direct view. The three sutures are used to stabilize the annulus of the implanted valve and to ensure circular expansion. Next, the AV is exposed via a conventional aortotomy. Severe calcification of the left coronary cusp is identified as the culprit for the gradient and the bioprosthetic dysfunction. Conventional replacement of the bioprosthesis seems to be risky due to the severe calcifications in the aortomitral curtain. The bioprosthetic leaflets are resected to avoid obstruction of the coronary ostia (*Figure 3B*). To fit in a larger aortic valve and to avoid PPM, the remaining bioprosthetic ring is cracked using a 22 mm ATLAS Gold Non-compliant balloon (*Figure 3C*). Subsequently, a 23 mm Edwards Sapien 3 valve is implanted under direct vision (*Figure 3D*). The total cross-clamp time is 76 min. Post-bypass echocardiography shows neither LVOT obstruction nor paravalvular leak or prosthesis dysfunction. The patient is extubated on the first post-operative day and transferred to the normal care unit. Transthoracic echocardiography before discharge reveals an adequate function of both prostheses. Systolic aortic valve mean gradient: 8 mmHg; aortic valve area: 2.35 cm^2^; systolic mitral valve mean gradient: 6 mmHg; indexed end-diastolic left ventricular volume: 31.14 mL/m^2^; indexed end-systolic left ventricular volume: 18.48 mL/m^2^; EF: 40.80%. There are no signs of paravalvular leak (PVL) or haemolysis. Long-term follow-up is needed to evaluate the clinical significance of the initial decline in EF after MV surgery.^8^

**Figure 4:**
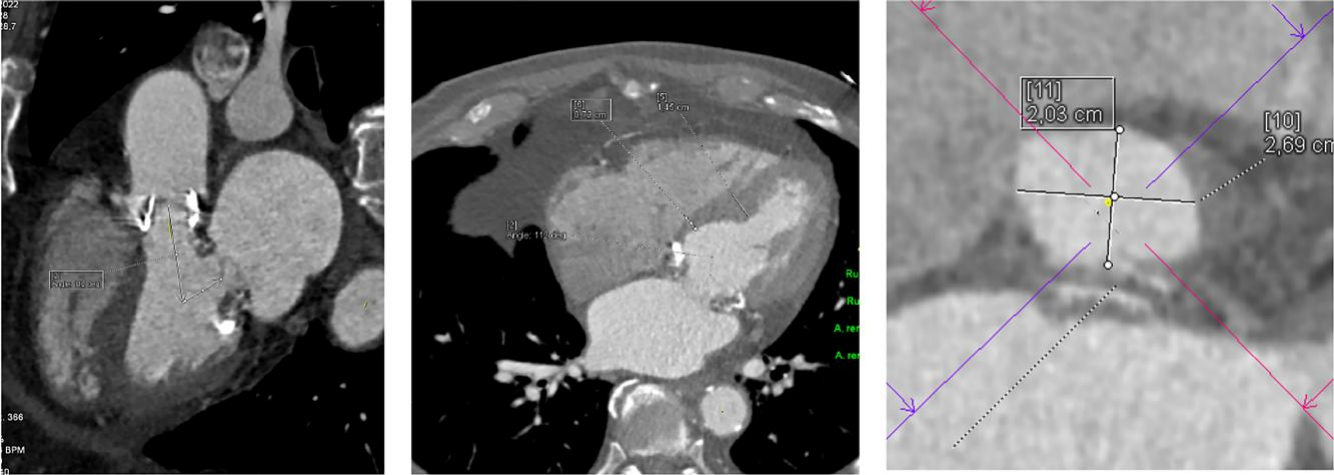
Post-operative CT scan.

The post-operative course is uneventful. The patient can be transferred to the post-operative IMCU on Day 1 and subsequently to the regular ward on Day 3. Post-operative CT confirms the adequate post-operative result (*Figure 4*). The patient is discharged one week after the intervention. The patient can do long walks and small hiking tours three months after the intervention without symptoms. Follow-up transthoracic echocardiography (TTE) reveals adequate prosthetic function, with an aortic effective orifice area of 2.35 cm^2^ and no signs of PVL.

## Discussion

We report an 82-year-old patient with double prosthesis degeneration six years AV and MV replacement. Different treatment strategies were discussed inter-disciplinary in the heart team with the patient and his family. Transcatheter double valve-in-valve replacement is a minimal-invasive treatment option for selected patients with double prosthesis regeneration but seemed unfeasible here due to LVOT and mitral annular calcifications. Therefore, the patient was evaluated for a transapical double valve implantation. However, due to the massive calcifications and an LV-inflow–outflow angle of >60°, the risk of LVOT obstruction seemed too high. Obstruction of neo-LVOT after transcatheter mitral valve replacement remains a major challenge,^9^ as it is associated with significantly higher procedural mortality. Estimated neo-LVOT area and aortomitral angulation are predictors for LVOT obstruction. An estimated neo-LVOT area ≤ 1.7 cm^2^ is considered high risk. Aortomitral angulation, usually measured in end-systole, describes the angle between the annular planes of the aortic and mitral valves. A near perpendicular orientation, where the mitral valve trajectory runs towards the septum, increases LVOT obstruction risk.^10^ Surgical re-double valve replacement was considered intensively. However, calcifications of the LVOT refrained us from exchanging the aortic valve. Moreover, extensive mitral annular calcification discouraged us from conventional MV re-replacement. Due to his surgical high-risk profile regarding age and concomitant severe mitral annular calcification, we decided to treat the patient with an open transcatheter double valve-in-valve replacement.

Mitral valve was exposed via the LA roof to optimize the trajectory path of implantation. Transatrial access for open transcatheter MV replacement has been suggested as a useful approach in patients with severe MAC.^11^ To avoid LVOT obstruction in our patient, we chose resection of the MV prosthesis leaflets with subsequent open implantation of the transcatheter valve. This is not feasible in a conventional transcatheter approach, where the displacement of the anterior MV (prosthesis) leaflet through the transcatheter valve can cause LVOT obstruction. One transcatheter option would have been the intentional laceration of the anterior mitral leaflet, called LAMPOON procedure (Intentional Laceration of the Anterior Mitral Leaflet to Prevent Left Ventricular Outflow Tract Obstruction During Transcatheter Mitral Valve Implantation). The results from a small cohort of patients are promising. This procedure is challenging, and patients have to be selected carefully, i.e. leaflets have to be free of calcification.^12^ Here, three sutures were placed at the ring of the degenerated mitral bioprosthesis corresponding to the three commissures to guarantee circularity of expansion and to avoid a post-operative paravalvular leak. Prior to the implantation of the aortic prosthesis, the leaflets of the aortic valve prosthesis were excised to avoid coronary obstruction. The remaining ring of the aortic valve was cracked to enable the implantation of a larger (23 mm) prosthesis to avoid PPM—a technique that has proven useful in using larger valve-in-valve prosthesis.^12^

This case adds valuable insights into outstanding issues in treating patients with valvular heart disease from a lifetime perspective. Ever-increasing survival rates after valvular procedures scale up the need for secondary procedures. The choice of the first intervention should be driven, amongst commonly known factors, by the options for re-intervention.

We herein conclude that open transcatheter double valve-in-valve replacement for degenerated bioprostheses on the arrested heart might be a valuable choice to treat selected high-risk patients with bioprosthetic valve degeneration.

## Data Availability

The data associated with this case report are available upon reasonable request.

## Lead author biography

Can Gollmann-Tepeköylü obtained a PhD in Molecular Cell Biology focusing on heart failure and valve disease. He has done research fellowships in the USA, Switzerland, and Taiwan and is part of a translational research group based in Innsbruck, Austria. The group was awarded numerous national and international research awards for their work on cardiac regeneration and valvular heart disease. Currently, he is a staff surgeon at the Department of Cardiac Surgery at the Medical University of Innsbruck. His areas of interest are mitral valve surgery, coronary revascularization, and heart failure surgery.

**Consent:** Informed consent was obtained from the patient following COPE guidelines.

**Funding:** None declared.
